# Risk of rupture of an aortorenal vein graft aneurysm after the surgical repair of Takayasu arteritis-induced right renal artery stenosis: A case report and a literature review

**DOI:** 10.1097/MD.0000000000017889

**Published:** 2019-11-27

**Authors:** Xiyang Chen, Bin Huang, Ding Yuan, Yi Yang, Jichun Zhao

**Affiliations:** Department of Vascular Surgery, West China Hospital of Sichuan University, Chengdu, Sichuan, China.

**Keywords:** Takayasu arteritis, renal artery stenosis, aortorenal bypass, aortorenal vein graft aneurysm

## Abstract

**Introduction:**

Takayasu arteritis (TA) is a chronic and nonspecific inflammatory disease mainly affecting the aorta and its major branches, resulting in the stenosis or occlusion of target arteries. Approximately 50% to 60% of patients with TA likely have renal artery stenosis (RAS), which results in refractory hypertension (HTN) and renal dysfunction. Aortorenal bypass with saphenous vein graft (SVG) is the classical procedure to relieve patients’ symptoms. Graft restenosis is the most common complication during long-term follow-up. However, aortorenal vein graft aneurysm (AVGA) is uncommonly reported, and symptomatic or ruptured AVGA that needs reoperation is even rarer. Long-term follow-up results after AVGA reoperation also remain scare. Here, we introduced the long-term result of a symptomatic AVGA under the reoperation of polytetrafluoroethylene (PTFE) graft replacement and provided a literature review of AVGA reoperation after surgical bypass for RAS.

**Clinical finding:**

An 18-year-old male complained about mild to severe right lumbar pain for 5 days. He underwent right aortorenal bypass with SVG for TA-induced right renal artery stenosis to relieve refractory HTN and renal dysfunction 2 years ago. However, this patient did not proceed with a follow-up after the procedure. Physical examination showed normal vital signs, and an obvious percussion tenderness over the right kidney region was detected. The updated computed tomography angiography (CTA) revealed a right AVGA with a maximum diameter of 26 mm. No restenosis of the proximal and distal anastomoses was detected.

**Diagnosis:**

The patient was diagnosed to have right aortorenal vein graft aneurysm at the risk of rupture and Takayasu arteritis.

**Interventions:**

The AVGA was resected with a 6 mm PTFE graft replacement. An end-to-side proximal anastomosis to the orifice of the original anastomosis on the abdominal aorta and an end-to-end distal anastomosis to the distal normal renal artery were made.

**Outcomes:**

The patient had an uneventful postoperative clinical course and was discharged from the hospital 5 days after the operation. The 4-year updated CTA revealed no restenosis or aneurysmal degeneration of the prosthetic graft.

**Conclusion:**

Symptomatic AVGA that needs reoperation is rare. Prosthetic graft replacement is an effective way to eliminate the risk of potential rupture. A 4-year satisfactory result indicative of a prosthetic graft can be the first choice for aortorenal bypass in RAS without active biological inflammation.

## 1 Introduction

Takayasu arteritis (TA) is a nonspecific inflammatory vasculitis that involves medium to large arteries, resulting in occlusive lesion or aneurysm degeneration. In 1908, Takayasu, a Japanese ophthalmologist, first described this disease in a young female patient with retinal neovascularization and absent radial pulses.^[1]^ Renal artery involvement causing stenosis or occlusive lesion occurs in up to 60% of patients with TA, resulting in refractory hypertension (HTN), renal dysfunction, and cardiac decompensation.^[2,3]^

Indications for revascularization procedures remain unclear because limited research has reported about the prognosis of patients who have TA with renal artery stenosis (RAS) and do not undergo vascular intervention. Revascularization procedures include endovascular therapy with or without stent and surgical repair with an autologous graft or a prosthetic graft.^[4–8]^ Endovascular therapy is the preferred initial treatment because it is minimally invasive. However, previous studies about endovascular therapy showed inconsistent results regarding long-term patency.^[4–6]^ In other studies, surgical bypass as an alternative has demonstrated superior patency rates to endovascular intervention. Aortorenal bypass with a saphenous vein graft (SVG) was previously the optimal option.^[7,8]^ One of the most common complications of surgery is the restenosis and dilation of SVG. However, reports on aortorenal vein graft aneurysm (AVGA) after surgical repair for RAS induced by TA are few, and ruptured AVGA is even rarer, with only 2 published cases without long-term results after reintervention.^[9,10]^ In this case, we first reported the long-term result of reintervention for AVGA at the risk of rupture and provided a literature review about the treatment of AVGA after surgical bypass for RAS.

## 2 Case report

**Figure 1:**
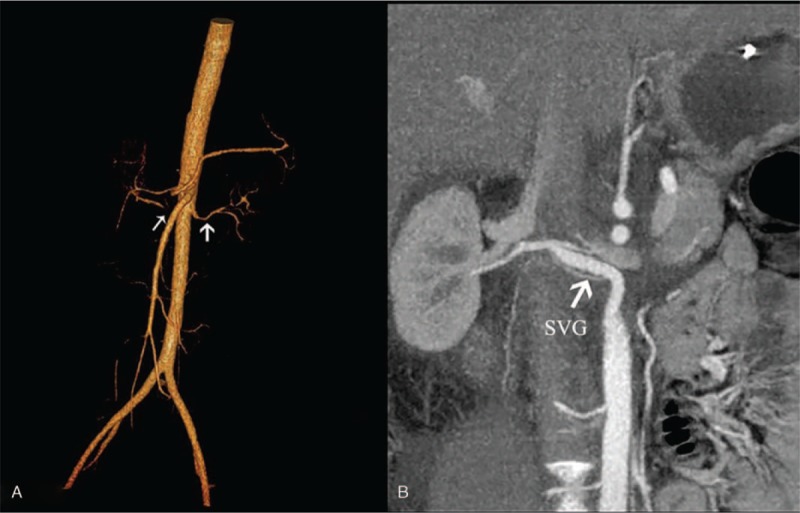
(A) CTA: Thin white arrow on the left indicates a severe stenosis lesion (>70%) in the middle segment of the right renal artery; thick white arrow on the right denotes a slender left renal artery. (B) CTA: Aortorenal bypass with SVG.

An 18-year-old male complained about refractory HTN for 2 years. He was under therapy involving 2 different antihypertensive medicines, and his blood pressure varied from 160/110 mm Hg to 150/100 mm Hg. Physical examination showed the following findings: temperature of 36.7°C, heart rate of 73 bpm, respiratory rate of 16 breaths per minute, and blood pressure of 162/90 mm Hg. Abdominal computed tomography angiography (CTA) revealed a severe stenosis lesion (>70%) in the middle segment of the right renal artery with obvious intimal hyperplasia in the stenosis part and slenderness of the left renal artery (Fig. 1A). His preoperative lab test revealed eGFR of 55 ml/minute, Crea of 59 μmol/L, CRP of 1.2 mg/L, and ESR of 8 mm/hour. Other immunological test results were negative. Based on the patient's symptoms and imaging features, a diagnosis of TA-induced right RAS with renovascular HTN and renal function impairment was made.

After the preoperative examination was completed, the right aortorenal bypass with SVG was surgically repaired through abdominal midline incision and transperitoneal exposure. Aortic inflow was located 3 cm below the level of the renal artery with a grossly normal lumen. Distal outflow was in the grossly normal renal artery bifurcation distal to the inflammatory process. The proximal and distal ends of the SVG were cut into an oblique section, and an end-to-side anastomosis was made for the renal artery with a saphenous vein segment. During the procedure, the clamping time of the infra-aorta was controlled within 25 minutes with systemic heparinization (0.5 mg/kg unfractionated heparin), and the blocked time for the right renal artery was limited to 20 minutes. The patient had an uneventful postoperative clinical course and was discharged from the hospital 4 days after the operation. One month after the procedure, eGFR increased to 95 ml/minute, and Crea remained normal. The blood pressure was well controlled by only 1 antihypertensive medicine. CTA showed that the right SVG was patent without any restenosis or dilation (Fig. 1B).

**Figure 2:**
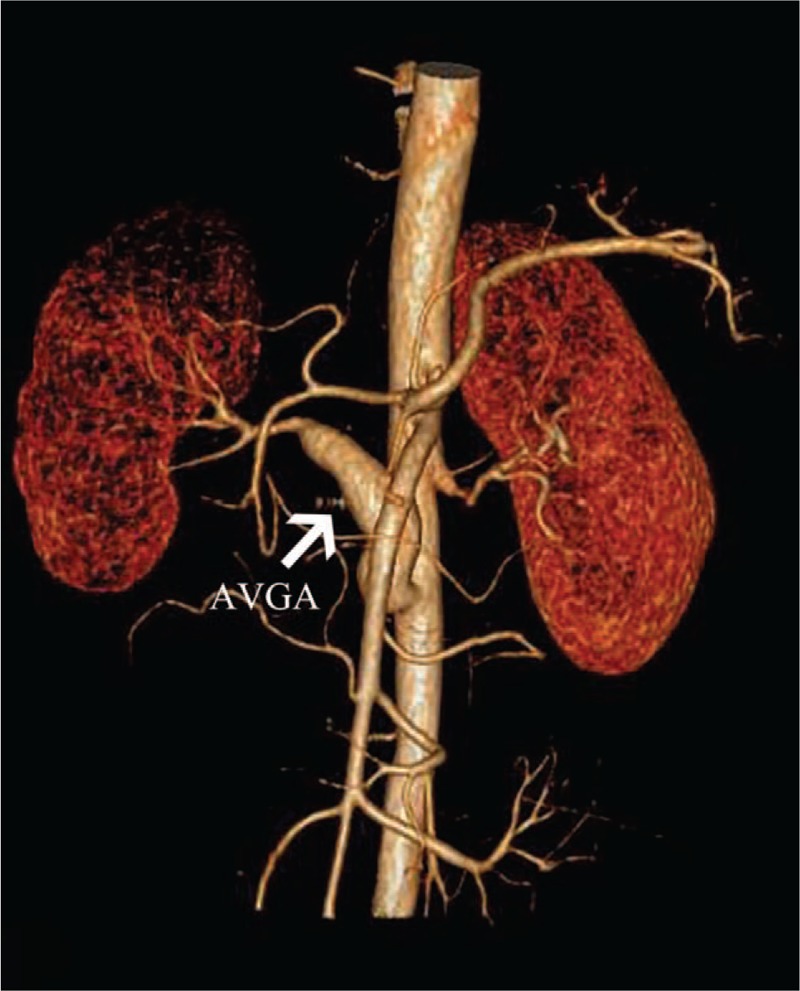
Two years after aortorenal bypass with SVG. The updated CTA revealed AVGA with a maximum diameter of 2.6 cm.

The patient did not proceed with the follow-up. Two years later, the patient returned to the emergency department for mild to severe right lumbar pain for 5 days. The updated CTA revealed a right AVGA with a maximum diameter of 26 mm. No restenosis of proximal and distal anastomoses was detected (Fig. 2). Physical examination showed the following findings: temperature of 36.6°C, heart rate of 74 bpm, respiratory rate of 16 breaths per minute, and blood pressure of 132/89 mm Hg. An obvious percussion tenderness over the right kidney region was detected. The updated lab tests indicated an eGFR of 98 ml/minute, a normal Crea level, a CRP of 1.1 mg/L, and an ESR of 18 mm/hour. Other immunological test results were negative.

**Figure 3:**
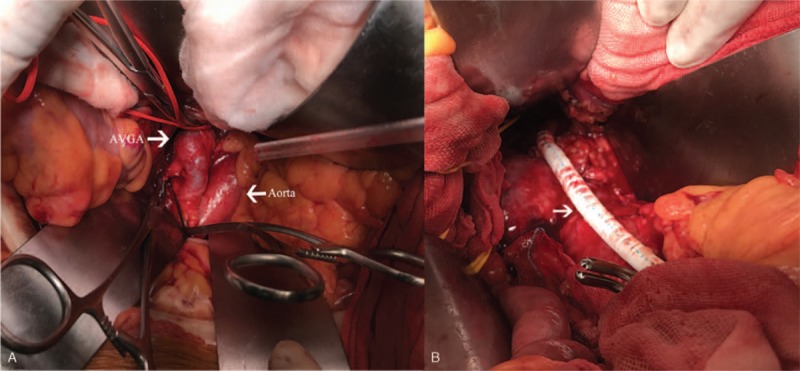
(A) Operative finding showed the aneurysmal formation of SVG (white arrow on the left). The abdominal aorta was normal (white arrow on the right). (B) AVGA was resected with the replacement of a PTFE graft (white arrow).

**Figure 4:**
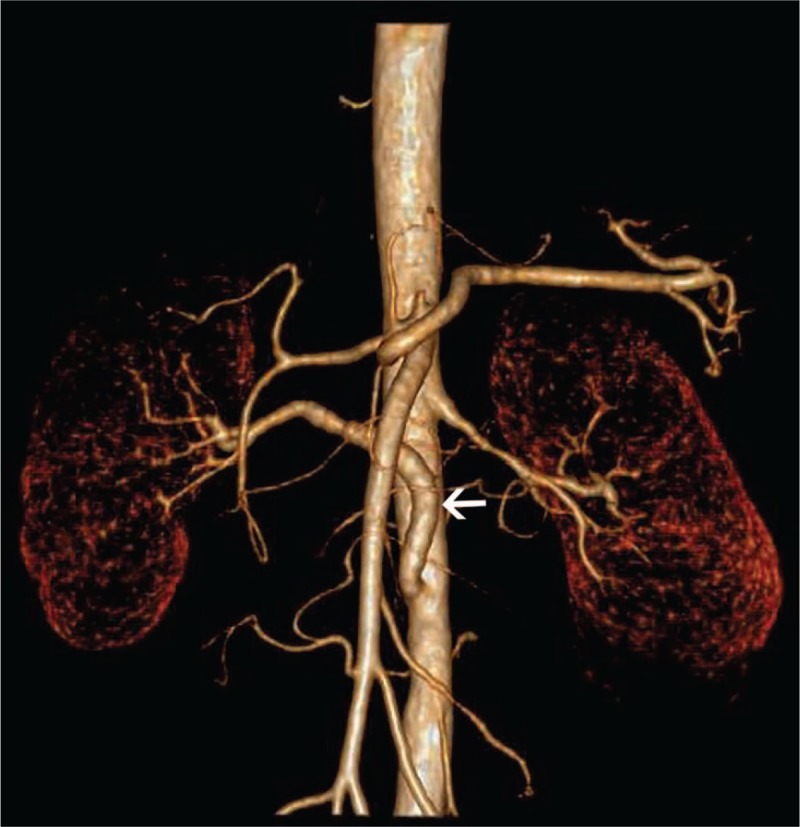
CTA: Four-year follow-up after reoperation revealed a normal PTFE graft (white) without restenosis or aneurysmal degeneration.

The diagnosis of a right AVGA at the risk of rupture was made. Though the maximum diameter of AVGA was not that large, AVGA was resected, considering the symptoms of mild to severe right lumbar pain. A 6 mm Gore polytetrafluoroethylene (PTFE) graft was interposed. An end-to-side proximal anastomosis to the orifice of the original anastomosis on the abdominal aorta and an end-to-end distal anastomosis to the distal normal renal artery were made (Fig. 3A and B). The patient had an uneventful postoperative clinical course and was discharged from the hospital 5 days after the operation. The right lumbar pain was completely relieved, and blood pressure was well controlled. The 4-year follow-up result showed that the blood pressure and the lab tests remained normal during follow-up. The updated CTA revealed no restenosis or aneurysmal degeneration of the anastomosiswas detected (Fig. 4).

## 3 Discussion

TA is a chronic and nonspecific inflammatory disease mainly affecting the aorta and its major branches, resulting in the stenosis or occlusion of target arteries. Approximately 50% to 60% of patients with TA likely have RAS, which results in refractory HTN, cardiac inefficiency, and renal dysfunction. The revascularization of the renal artery may be necessary when symptoms worsen. Though revascularization procedures include endovascular therapy with or without stent deployment and surgical repair, studies have reported that surgical revascularization has a long-term patency and a lower risk of restenosis compared with those of endovascular therapy. Otherwise, surgical repair is more suitable for long segments and severe stenosis and have a broader indication than endovascular therapy.^[7,8,11]^

The reconstruction of the renal artery with the saphenous vein and a prosthetic material as a bypass graft is durable for atherosclerotic disease. However, the risk of SVG dilation in pediatric patients and patients without atherosclerosis remains unresolved. For the long-term patency of an aortorenal bypass graft, most studies have focused on SVG complications or prosthetic graft restenosis and SVG dilation. Limited studies have described AVGA, especially symptomatic or ruptured AVGA. Rarer cases of AVGA reoperation and long-term results after re-intervention have been reported because most cases of AVGA remain stable during follow-up and are uneventful.^[12–17]^ In 1973, Stanley first reported the follow-up results of 100 cases of aortorenal SVG, indicating that SVG has a higher likelihood of dilation or aneurysmal degeneration in a pediatric population; a long-term follow-up is necessary for the assessment development of SVG.^[13]^ However, in a review about the complications of renal artery reconstruction that requires reoperation, the aneurysmal deterioration of vein grafts is an uncommon reason, accounting for only 3 (1%) of 278 aortorenal saphenous vein bypass grafts requiring revision.^[14]^ Dean also reported that about 20% (8/39) of the aortorenal SVG dilates, whereas AVGA is detected in only 2 patients without growth during follow-up.^[15]^ In a report on anastomotic aneurysms after the surgical repair of TA, the incidence of anastomotic aneurysm in 103 patients with surgically treated TA is 8.5%. However, aneurysm degeneration involves anastomosis, not saphenous vein bypass. Otherwise, the involved arteries are the aorta, the subclavian artery, and the carotid artery; furthermore, 25% lesions of aneurysms in TA indicate that aneurysmal TA is an independent predictor of the development of an anastomotic aneurysm.^[16]^

The aneurysm formation of SVG in non-aneurysmal renal artery lesion caused by TA is extremely rare. Cases requiring a reoperation are even rarer, and only 2 cases provided 1- and 2-year follow-up results after the second intervention.^[18,19]^ Other cases of AVGA after surgical bypass for RAS are either under observation without reoperation or under reoperation without follow-up after discharge. Our case underwent a 4-year follow-up with a satisfactory result. We searched relevant information about reoperations for AVGA after the surgical bypass of RAS via PubMed. The detailed information of the related studies is shown in Table 1.

**Table 1:**
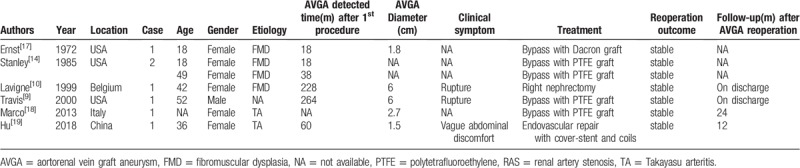
Literatures information of AVGA reoperation after surgical bypass for RAS.

The most common way to solve AVGA is AVGA replacement by using a prosthetic graft. Five cases of AVGA were successfully resolved by aneurysm resection with an interposed prosthetic graft (4 PTFE, 1 Dacron). However, only 1 case documented a 1-year follow-up with a satisfactory result; 4 other cases did not reveal good results after discharge.^[17–20]^ We also adopted PTFE graft replacement. Excluding the PTFE graft, Bath reported the replacement of a previous AVGA by using a Dacron graft in 2012. In contrast to our procedure.^[21]^ An alternative autologous graft to SVG has also been proposed. Patterson reported the reconstruction of the renal artery with the radial artery and documented a satisfactory result in a case.^[22]^ Excluding surgical repair for AVGA, Hu also introduced a novel endovascular intervention for AVGA with a cover stent and coils.^[19]^ Berkowitz adopted a new way to prevent the risk of the aneurysmal degeneration of SVG. Our results showed that a tubular SVG supported by an external Dacron mesh could be a suitable graft material for renal reconstruction in a pediatric population. With an average follow-up of 4.3 years, no AVGA was detected in 19 mesh-supported grafts.^[23]^

Considering the satisfactory result after the 4-year follow-up, we believe that prosthetic graft may be the first choice of graft for patients who have TA-induced RAS and need to undergo aortorenal bypass if no active biological inflammation is detected because biological inflammation increases the likelihood of complications after aortorenal bypass in patients with TA.^[24]^

AVGA after surgical bypass with SVG for TA-induced RAS is uncommon. Symptomatic AVGA requiring a reoperation is even rarer. Prosthetic graft replacement is an effective way to eliminate the risk of potential rupture. However, the long-term result of reoperation for AVGA with prosthetic graft is still scarce. Our case demonstrated a satisfactory 4-year follow-up prosthetic result without restenosis or anastomotic aneurysmal formation, indicating that prosthetic graft might be considered as the first choice for RAS if no active biological inflammation was detected.

## Author contributions

**Conceptualization:** Jichun Zhao.

**Data curation:** Bin Huang.

**Resources:** Yi Yang.

**Software:** Yi Yang.

**Visualization:** Ding Yuan.

**Writing - original draft:** Xiyang Chen.

**Writing - review & editing:** Bin Huang, Jichun Zhao.
